# Relative Determination Approach to the Metabolites of Protoberberine Alkaloids in Rat Urine by Liquid Chromatography Tandem Mass Spectrometry for the Comparative Studies on Rhizome coptidis and Zuojinwan Preparation 

**Published:** 2012

**Authors:** Rui Yan, Qier Mu, Yin Wang, Youping Liu, Xin Di

**Affiliations:** *School of Pharmacy, Shenyang Pharmaceutical University, Shenyang, China. *

**Keywords:** Metabolite, Determination, High performance liquid chromatography, Tandem mass spectrometry, *Rhizome coptidis*, Zuojinwan preparation

## Abstract

The lack of authentic standards has limited the quantitative analysis of herbal drugs in biological samples. The present work demonstrated a practicable strategy for the assay of herbs and their metabolites independent of authentic standards. A liquid chromatography– electrospray ionization–mass spectrometry (LC–ESI–MS) method for the qualitative and quantitative determination of the metabolites after oral administration of *Rhizome coptidis *and Zuojinwan preparation in rat urine has been developed. Urine samples, extracted with a protein precipitation procedure were separated on a C_18 _column using a mixture of water (containing 0.1% formic acid) and acetonitrile (30:70, v/v) as mobile phase. The detection was performed via MS with electrospray ionization interface in positive selected reaction monitoring (SRM) mode. One urine sample after administration was selected as ‹standard›. The method validation was carried out according to a conventional method which was calibrated by authentic standards. The fully validated method was applied to the pharmacokinetic study of 2,9-demethyljateorhizine-3-sulfate, 13-methoxyjateorhizine-3- glucoronide and 6-methyljateorhizine-5-glucoronide in rat urine. The results could provide evidence to explain the combination of *Rhizome coptidis *and *Evodiae fructus *in terms of elimination.

## Introduction

Traditional Chinese medicine (TCM) mainly uses combinations to produce new pharmacological activities through a synergistic effect or antagonistic action. Studies on the interaction between herbs are useful for probing the mechanism of TCM (1-3). 

In the clinical practice of TCM, Zoujinwan preparation, which consists of *Rhizome coptidis *- *Evodiae fructus *powder (6:1, g/g), has been used to treat gastro-intestinal disorders with a long history (4). *Rhizoma coptidis *is widely used in TCM due to its broad therapeutic effects (5-7). There were several investigations on the disposition and metabolism of *Rhizoma coptidis *using high performance liquid chromatography-tandem mass spectrometry (8-11). As far as the authors know, there was no method published for simultaneous determination of metabolites in biological fluids after oral administration of *Rhizoma coptidis *and Zuojinwan preparation.

Scarcity of standards for calibration seriously impedes the extensive analysis of the constituents of TCM, especially the studies of metabolites. Due to very low concentrations and complex situation, metabolites were usually hard to be separated from the biological system. As a result, previous studies on metabolites always focused on quality analysis (12). Little information is available related to the quantity of metabolites. This paper designed an alternative analytical approach to the relative quantity of metabolites in biological samples. A urine sample of the rat after administration was selected as ‘standard’ to develop a fully validated method. On this basis, the excretion kinetic differences of metabolites between the single herb and the whole prescription could be compared with this relative determination.

## Experimental

*Chemical and reagents*

Methanol, formic acid, and acetonitrile were of chromatographic grade from the Yuwang Chemical Factory (Shandong, China). Deionized water was purified using an Alpha-Q water-purification system (Millipore, Bedford, MA, USA) for the preparation of samples and buffer solution. All other reagents were of analytical grade. *Rhizoma coptidis *and *Evodia rutaecarpa *were purchased from the Sifang Pharmacy (Shenyang, China).

*Pharmacokinetic study*

Six male Sprague-Dawley rats (250 ± 20g) were fasted for 12 h prior to experiment. The rats were divided into two groups to complete the crossover design for pharmacokinetic experiment with a washout period of 7 days. The powder of *Rhizome coptidis *and Zuojinwan preparation was suspended in 0.1% carboxymethyl cellulose sodium (CMC-Na) aqueous solution and was administered to the rats (1.08 g *Rhizome coptidis *and 0.18 g *Evodia rutaecarpa *powder/kg body weight) by oral gavage. Urine samples were collected within 0-24 h and 24-48 h following administration, measured the volume of each sample accurately and stored at -20°C for preservation. *Quality analysis*

*Apparatus and operating conditions*

Qualitative analysis was operated on a ThermoFinnigan LCQ linear ion-trap mass spectrometer (ThermoFinnigan, San Jose, CA, USA) fitted with an electrospray ionization source over the mass range from *m/z *50 to 2000 in the positive ionization mode. Xcalibur 1.2 data analysis system was used. The spray voltage was set to 4.2 kV. The capillary voltage was fixed at 13 v. The heated capillary temperature was fixed at 200°C. Nitrogen used as the sheath and the auxiliary gas was set to 70 and 20 arbitrary units, respectively. The isolation width for MSn was 1.0 Da. The HPLC system consists of an Agilent 1100 series equipped with an Agilent 1100 series photodiode-array detector (PDA) and autosampler Data analysis (Agilent, Palo Alto, CA). Chromatographic separation was carried out on a Diamonsil C18 (150 × 4.6 mm I.D., 5 μm, Dikma) with an EasyGuard C18 Security guard column (8 × 4.0 mm I.D., Dikma). The mobile phases consisted of 0.3% formic acid (A) and acetonitrile (B) using a gradient elution of 20% B at 0 min, 45% B at 25 min, 45% B at 40min, at a flow rate of 0.5 mL/min. The column temperature was 30°C, detection wavelength was at 245 nm and the injection volume was 20 μL.

*Sample preparation*

Urine samples were filtered through 0.45 μm micro membrane (Truelab Co. Shanghai). The filtrate was passed through C18 solid-phase extraction cartridges (200 mg / 3 mL) (Waters Co., Milford, MA) that had been activated with 2 mL of MeOH and equilibrated with 2 mL of water. The constituents were washed with 1 mL water and eluted with 2 mL of MeOH from the cartridge, and then the eluate was evaporated under a stream of nitrogen at 45°C to leave a residue that was dissolved in 200 μL of mobile phase for LC/MSn analysis.

*Quantitative analysis*

*Apparatus and operating conditions*

The HPLC system consists of a LC-10ADvp Pump (Shimadzu, Kyoto, Japan) and a SIL-HTA Autosampler (Shimadzu, Kyoto, Japan). Chromatographic separation was carried out on a Diamonsil C18 (150×4.6 mm, 5μm, Dikma) column with a EasyGuard C18 Security guard column (8×4.0 mm I.D., Dikma) kept at 20°C. The mobile phase consists of water (containing 0.3% formic acid)/acetonitrile (30:70, v/v), at a flow rate of 0.45 mL/min. The injection volume was 10 μL.

Mass spectrometric detection was performed on a Thermo Finnigan TSQ Quantum triple quadrupole mass spectrometer (San Jose, CA, USA) equipped with an ESI source in the positive ionization mode. The MS operating conditions were optimized as follows: the spray voltage: 4200 v; the heated capillary temperature: 270°C; the sheath gas (nitrogen): 30 Arb; the auxiliary gas (nitrogen): 5 Arb; the collision gas (argon) pressure: 1.2 mTorr. Data acquisition was performed by Xcalibur 2.0 software. Peak integration and calibration were performed using LCquan software. Quantification was obtained by using SRM mode of the transitions at *m/z *390→310 for M1, at *m/z *542→366 for M2, at *m/z *544→368 for M3 and at *m/z *172→128 for metronidazole (IS) respectively, with a scan time of 0.3 sec per transition.

*Standard solution and quality control samples preparation*

No. 3 rat urine 0-24 h after oral administration of Zuojinwan preparation acted as standard stock urine with an assumptive concentration of c for each analyte. Working solutions of the analytes were prepared by spiking the blank rat urine with 5, 10, 20, 50, 100, 150, 200 μL of the standard stock urine, respectively to yield the following concentrations: 0.025, 0.05, 0.1, 0.25, 0.5, 0.75, 1.0 c. Quality control (QC) samples were prepared from blank urine at concentrations of 0.05, 0.25, 0.75 c. The working solution of internal standard (IS), at 200 ng/mL was prepared by diluting metronidazole stock solution (200 μg/mL) with methanol. All the solutions were stored at -20°C.

*Sample preparation*

To 200 μL rat urine in a 1.0 mL eppendorf tube, 50 μL of the internal standard solution (200 ng/mL), 800 μL of acetonitrile were added. This mixture was vortex-mixed 2 min and centrifuged at 2148 × g for 5 min. The supernatant was separated out and blown to dryness with nitrogen at 40°C. Then the residue was reconstituted in 100 μL mobile phase and mixed to make final testing samples. A 10 μL aliquot of the final testing samples was injected onto the LC-MS/MS system for analysis.

*Method validation*

The selectivity was investigated through preparing and analyzing six different batches of blank rat urine samples to ensure the absence of endogenous compounds with the same retention times as metabolites and internal standard.

Calibration standards of seven analytes concentration levels were extracted and assayed. The analytes calibration curve was generated by plotting the peak-area ratios of analytes to the IS (y) versus the concentrations of analytes (x), using weighed least squares linear regression (the weighing factor was 1/x2). The LLOQ for each analyte in urine was defined as the lowest concentration at which both precision and accuracy were less than or equal to 20%.

Accuracy and precision were investigated by determining LLOQ and QC samples at three concentration levels of 0.025, 0.05, 0.25, 0.75 c (six samples for each concentration level) on 3 different validation days. The concentration of each sample was calculated using a calibration curve constructed on the same testing day. Accuracy was described as relative error (RE) and precision was described as relative standard deviation (RSD). The criteria used to assess the suitability of precision and accuracy was as follows: the RSD should not exceed 15% and the accuracy should be within 15% of the actual value for QC samples.

To determine extraction recovery, extracted samples were prepared through the following procedure: QC samples at three concentration levels of 0.05, 0.25, 0.75c (three samples for each concentration level) were processed according to the “Sample preparation”; half of these processed QC samples were reconstituted in blank urine 200 μL, and then were processed according to the “Sample preparation”. The extraction recoveries of the analytes were determined by comparing the mean peak areas of six re-extracted low (0.05 c), medium (0.25 c) and high (0.75 c) samples to mean peak areas of six extracted samples at the same concentrations. Recovery of IS was also evaluated by comparing the mean peak areas of six extracted medium samples to mean peak areas of six reference solutions spiked in extracted plasma samples of the same concentration.

By exposing QC samples to different temperature conditions for different periods of time, the stability of analytes was investigated at two concentration levels of 0.05 and 0.75 c (three samples for each concentration level). The stability studies included: (a) stability at room temperature for 4 h; (b) stability after three freeze–thaw cycles; (c) stability of the extracted samples at room temperature for 24 h.

The matrix effect (ME) was examined by comparing the peak areas of the metabolites between two different sets of samples. In set 1, QC at middle concentration was processed according to “Sample preparation”. These analyses were repeated six times. In set 2, six different batches of blank rat urine samples were processed according to the “Sample preparation”. The residue was reconstituted in 50 μL stock standard urine and 150 μL blank urine. The mixture was processed according to “Sample preparation”. Ratio of the mean peak areas of set 2 to that of set 1 would indicate the possibility of ionization suppression or enhancement for analytes and IS. If the ratio is less than 85% or more than 115%, an exogenous matrix effect is implied. The assessment of the relative ME was made by a direct comparison of the analyte peak area values between different sources of urine. The ME of internal standard was assessed by comparing the peak area of its working solution added into the extract of precipitated blank urine with the peak area of the working solution. 

## Results and Discussion

*Quality analysis*

LC-MSn was used for the qualitative analysis of the metabolites after administration of herb powders. The mobile phase was selected to optimize the separation and ionization efficiency. Three metabolites were found in both urine samples after administration of *Rhizome coptidis *and Zuojinwan preparation. Their molecular weights were concluded on the basis of their positive ion electrospray mass spectra, which showed precursor ions (Table 1). 

**Table 1:** Precursor and product ions of metabolites in the rat urine in LC-MSn experiments

**Metabolites**	***m/z***	**MS** **2**	**MS** **3**	**MS** **4**	***t*** **R** **(min)**	**Formula**	**Identification**
M1	390	310	295	267	11.01	C18H16NO7S+	2,9-demethyljateorhizine-3-sulfate
M2	542	366	320	292	10.79	C27H28NO11+	13-methoxyjateorhizine-3-glucoronide
M3	544	368	322	307	8.03	C27H30NO11+	6-methyljateorhizine-5-glucoronide

**Figure 1:**
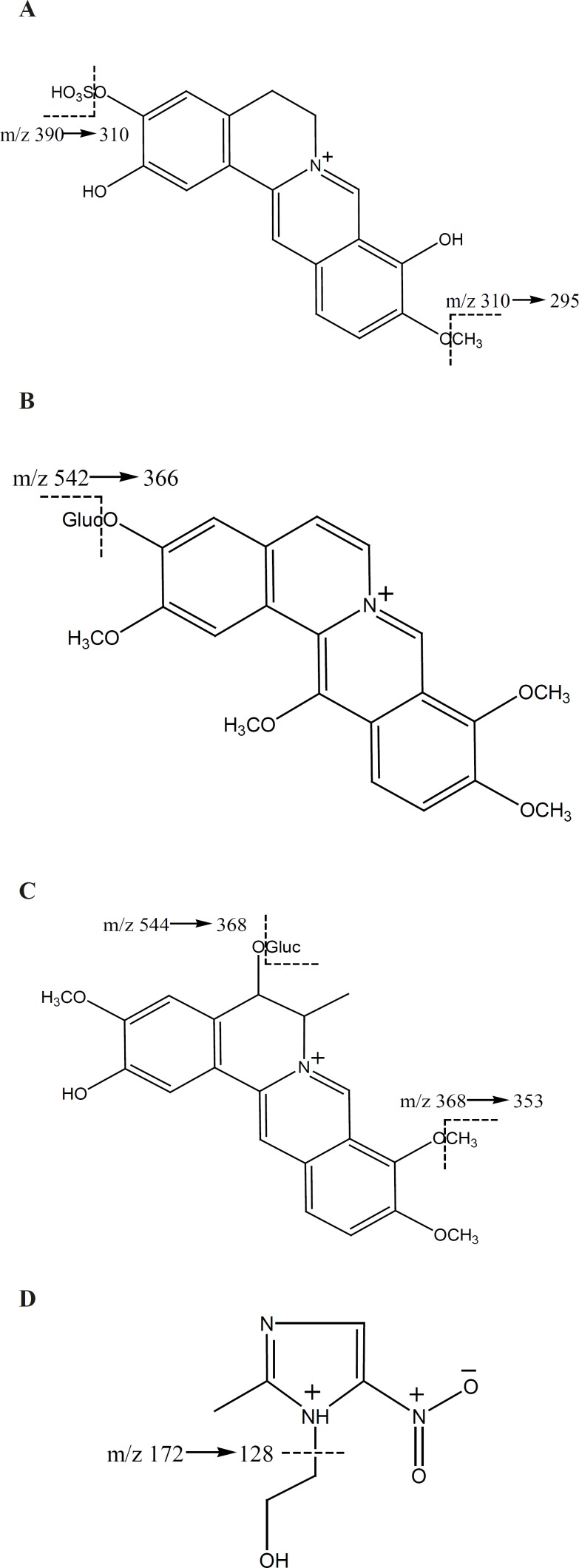
Chemical structure of (A) M1 (2,9-demethyljateorhizine- 3-sulfate) (B) M2 (13- methoxyjateorhizine-3-glucoronide) (C) M3 (6-methyljateorhizine-5-glucoronide) and (D) IS (meteronidasole0.

M1 and M2 have not been reported previously. The possible structures of the three metabolites were deduced by careful studies on their MS and MSn spectra and by referring to literature data (8, 9, 13). The deduced chemical structures of the metabolites are shown in Figure 1. Protoberberine alkaloids are the parent alkaloids of the three metabolites. They are a group of constituents with similar structures and usually considered as the most important pharmacologically active constituents in *Rhizome coptidis*. Studying their metabolites is useful for understanding the disposal of active constituents in-vivo.

*Quantity analysis*

*Optimization of LC-MS/MS*

**Figure 2:**
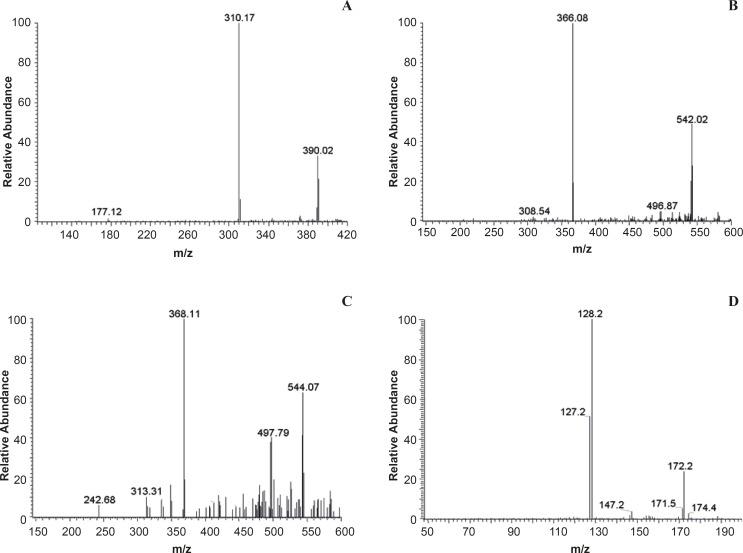
Product ion mass spectra of [M+H]+ ions of (A) M1 (2,9-demethyljateorhizine-3-sulfate) (B) M2 (13- methoxyjateorhizine-3-glucoronide) (C) M3 (6-methyljateorhizine-5-glucoronide) and (D) IS (meteronidasole

The typical MS2 full-scan mass spectra of metabolites and IS is described in Figure 2.

**Figure 3:**
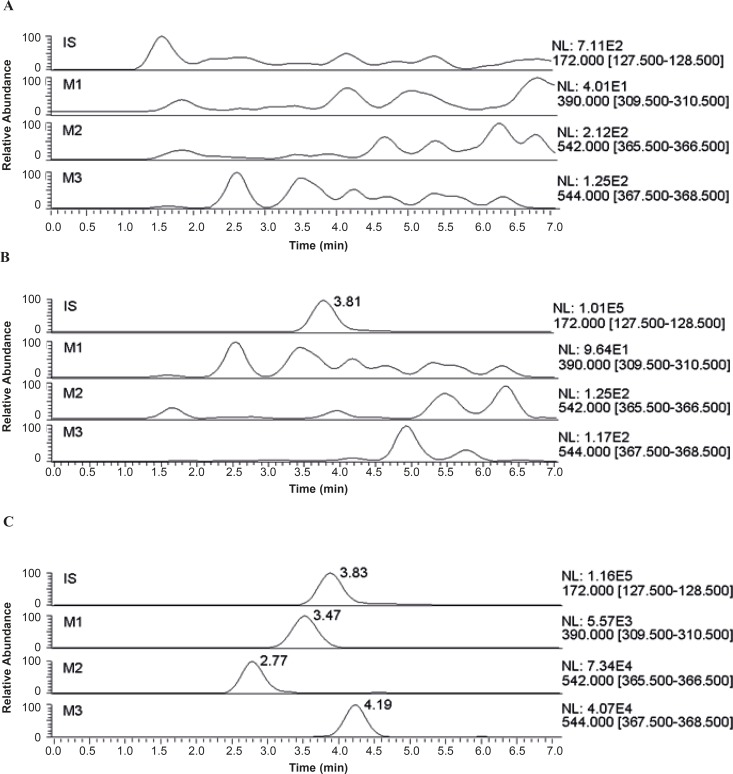
Representative SRM chromatograms for metabolites in (A) a blank urine (B) a blank urine spiked with IS (C) a urine sample after administration of Zuojinwan preparation

 The most abundant fragments in the product ion full-scan spectra of the substances were elected as the SRM transitions. To obtain maximum sensitivity of the SRM, some parameters such as spray voltage, capillary temperature, source CID, sheath gas (nitrogen) pressure, auxiliary gas (nitrogen) pressure, collision gas (argon) pressure, and collision energy were optimized. The other MS parameters were adopted from the recommended values for the instrument. The selected mobile phase provided low background noise and proper retention time. The typical chromatograms of blank urine and a urine sample 0~24 h after oral administration were presented in Figure 3. All samples were found to be of no interference at the retention times of the analytes or the IS. 

*Method validation*

The process of sample preparation may introduce errors to the determination which can be corrected with the use of an internal standard method. As internal standard, metronidazole does not exist in herb extract and functions through an ion channel different from analytes. After separating by HPLC, the retention time of metronidazole was in the middle of analytes. In addition, its recovery rate was 81.5%, which was generally consistent with analytes and thus ensured the ideal result. 

Typical equations of the calibration curve were listed in Table 2 and showed excellent linearity. The precision and accuracy data corresponding to LLOQ are shown in Table 3. 

**Table 2:** The regression equations for the metabolites in rat urine

**Analyte**	**Linear model**	***r*** **2**	**Range**	**LLOQ**
**M1**	y = 0.000354+0.0545x	0.9870	0.050c~1.000c	0.050c
**M2**	y = -0.00563+0.767x	0.9804	0.025c~1.000c	0.025c
**M3**	y = 0.0326+0.822x	0.9837	0.050c~1.000c	0.050c

**Table 3:** The precision and accuracy for the metabolites in rat urine (n = 6).

**Analyte**	**Added (c)**	**Found (c)**	**RSD%**		**Relative error**	**Recovery %**	
			**Intra-day**	**Inter-day**	**%**	**mean**	**SD**
**M1** **M2** **M3** **IS**	0.050	0.05	15.5	18.4	4.2	79.7	13.6
	0.250	0.22	12.8	14.9	13.3	72.6	13.6
	0.750	0.76	7.7	12.2	-1.2	84.0	13.4
	0.025	0.02	14.6	/	0.4	/	
	0.050	0.05	10.1	4.2	4.4	74.7	12.2
	0.250	0.20	9.8	11.2	18.3	78.8	7.8
	0.750	0.77	9.3	14.2	-2.5	80.8	6.3
	0.050	0.04	16.7	11.4	13.0	87.9	10.5
	0.250	0.13	12.6	10.2	6.7	80.0	12.1
	0.750	0.67	11.3	8.1	10.4	80.2	8.4
	200 ng/mL	/	/	/	/	81.5	6.0

Table 3 contained the intra- and inter-day precision and accuracy data for 3 metabolites. Most values of accuracy and precision were within recommended limits. The relatively high RSD maybe caused by excessive endogenous substances in biological samples, when the urine sample acted as standard. 

The average extraction recoveries determined for the three analytes were consistent, precise and repeatable. Data were shown in Table 3. 

Table 4 summarized the stability data of QC samples. The results showed that all the samples were stable during these tests and there were no stability related problems during the routine analysis of samples for the pharmacokinetic study. 

**Table 4:** The stability for the constituents and metabolites in rat urine (n = 3).

**Analyte**	**Concentration (c)**	**Short-term**				**Three freeze-thaw**			**Post-preparative**	
		**Mean (c)**	**RSD%**	**RE%**	**Mean (c)**	**RSD%**	**RE%**	**Mean (c)**	**RSD%**	**RE%**
**M1**	0.05	0.05	23.6	-6.9	0.05	5.1	8.88	0.05	12.9	1.8
	0.75	0.80	6.4	6.2	0.79	12.1	5.2	0.63	4.9	-16.5
**M2**	0.05	0.05	14.4	3.2	0.04	4.5	-14.4	0.06	1.6	11.4
	0.75	0.70	7.9	-6.3	0.71	14.8	-5.5	0.77	8.1	2.3
**M3**	0.05	0.05	7.1	6.8	0.05	0.4	-6.6	0.05	8.2	6.8
	0.75	0.74	22.8	-1.2	0.70	12.6	-6.7	0.63	10.6	-16.3

The data displayed in Table 5 indicated that endogenous substances slightly suppressed the ionization of M1 under the present chromatographic and extraction conditions when the ESI interface was utilized. The low RSD value of absolute ME in six different sources of rat urine indicated that the relative ME for the analytes were minimal in this study. The ionization suppression/enhancement of M2, M3 and IS was negligible. 

**Table 5:** The matrix effect for the metabolites and IS in rat urine (n = 6).

**Analyte**	**Nominal concentration**	**Matrix effect %**	**RSD %**
M1	0.25c	67.48	2.6
M2	0.25c	92.87	19.1
M3	0.25c	86.31	11.7
IS	200 ng/mL	109.40	10.2

*Application to pharmacokinetic studies in rats*

**Figure 4:**
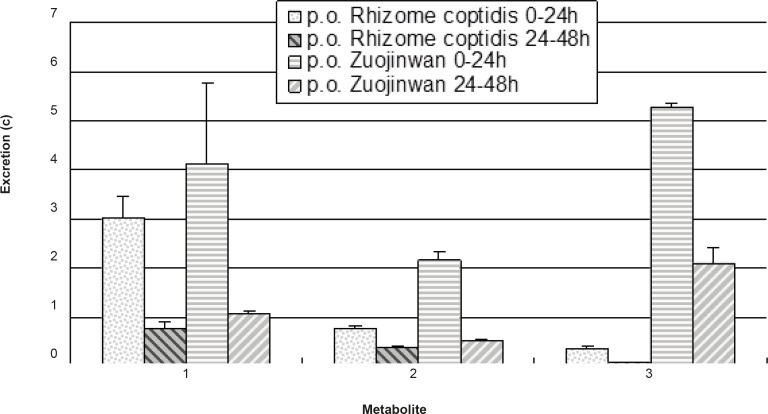
Excretion of the metabolites in rat urine (n = 6).

The validated analytical method was applied to the assay of metabolites in rat urine after oral administration of *Rhizoma coptidis *and Zuojinwan preparation. The excretion amount for each analyte can be determined according to the following equation: the volume of the urine samples (mL) × determined concentration (c). The results were presented in Figure 4.

More amount of metabolites were eliminated within 0-24 h than within 24-48 h, which indicated higher rate of elimination in 0-24 h than in 24-48 h; More amount of metabolites of *Rhizoma coptidis *were eliminated after coupling with *Evodiae fructus *in Zuojinwan preparation than before, which suggested that *Evodiae fructus *could promote the metabolism and elimination of the components in *Rhizoma coptidis*. As a result, *Evodiae fructus *plays the role of moderating the effect of *Rhizoma coptidis *in Zuojinwan preparation (14). One- way analysis of variance was applied to the comparison of the results before and after coupling: 0-24 h: F0.05 (1, 4) = 1.73 < 7.71, there is a significant difference; 24-48 h: F0.05(1, 4) = 2.48 < 7.71, there is a significant difference. In conclusion, combination could significantly affect the elimination of the three metabolites within different periods.

Metabolism is so important for clinical study. The ability of the assay to simultaneously quantitate metabolites can provide information regarding the concentration versus time profile to explain the formulation, the most appropriate dose and route of administration for TCM. The quantitative method developed in this study is useful for investigating the combination of *Rhizoma coptidis *and *Evodiae fructus *in Zuojinwan preparation. 

## References

[1] Luo XB, Chen B, Yao SZ (2005). Simultaneous analysis of protoberberine, indolequinoline and quinolone alkaloids in coptis–evodia herb couple and the Chinese herbal preparations by high-performance liquid chromatography-electrospray mass spectrometry. Talanta.

[2] Feng J, Xu W, Tao X, Wei H, Cai F, Jiang B, Chen WS (2010). Simultaneous determination of baicalin, baicalein, wogonin, berberine, palmatine and jatrorrhizine in rat plasma by liquid chromatography-tandem mass spectrometry and application in pharmacokinetic studies after oral administration of traditional Chinese medicinal preparations containing scutellaria-coptis herb couple. J. Pharm. Biomed. Anal.

[3] Adams M, Berset C, Kessler M, Hamburger M (2009). Medicinal herbs for the treatment of rheumatic disorders-A survey of European herbals from the 16th and 17th century. J. Ethnopharmacol.

[4] Shen YJ (2000). Pharmacology of Traditional Chinese Medicine.

[5] Tanaka T, Metori K, Mineo S, Hirotani M, Furuya T, Matsumoto H, Satoh T, Kobayashi S (1991). Studies on collagenase inhibitors. IV. Inhibitors of bacterial collagenase in Rhizome coptidis. Yakugaku Zasshi.

[6] Bova S, Padrini R, Goldman WF, Berman DM, Cargnelli G (1992). On the mechanism of vasodilating action of berberine: possible role of inositol lipid signaling system. J. Pharmacol. Exp. Ther.

[7] Chiou WF, Yen MH, Chen CF (1991). Mechanism of vasodilatory effect of berberine in rat mesenteric artery. Eur. J. Pharmacol.

[8] Pan JF, Yu C, Zhu DY, Zhang H, Zhang JF, Jiang SH, Ren JY (2002). Identification of three sulfate-conjugated metabolites of berberine chloride in healthy volunteers› urine after oral administration. Acta Pharmacol. Sin.

[9] Qiu F, Zhu ZY, Piao SJ, Zhang JY, Yao XS (2005). Studies on the metabolites in human urine after oral administration of berberine hydrochloride. Chin. Med. Res. C1in.

[10] Zuo F, Nakamura N, Akao T, Hattori M (2006). Pharmacokinetics of berberine and its main metabolites in conventional and Pseudo germ-free rats determined by liquid chromatography/Ion trap mass spectrometry. Drug Metab. Dispos.

[11] Han FM, Zhu MM, Chen HX, Chen Y (2006). Liquid chromatography-tandem electrospray ionization ion trap mass spectrometric assay for the metabolites of jatrorrhizine in rat urine. Acta Pharm. Sinica.

[12] Nassar AF, Talaat RE (2004). Strategies for dealing with metabolite elucidation in drug discovery and development. Drug Discovery Today.

[13] Wang DW, Liu ZQ, Guo MQ, Liu SY (2004). Structural elucidation and identification of alkaloids in Rhizoma coptidis by electrospray ionization tandem mass spectrometry. J. Mass Spectrom.

[14] Deng YT, Liao QF, Bi KS, Yao MC, Jiang XF, Xie ZY (2008). Studies on the dissolution rules of the main components of Coptis-Evodia herb couple. Chin. Trad. Patent Med.

